# Unlocking 1,3-Propanediol Production by *Pseudomonas aeruginosa* through Electro-Fermentation

**DOI:** 10.1021/acsomega.5c05374

**Published:** 2025-09-22

**Authors:** Julia Pereira Narcizo, María-Eugenia Guazzaroni, Adalgisa Rodrigues de Andrade, Valeria Reginatto

**Affiliations:** † Department of Chemistry, Faculty of Philosophy, Sciences and Letters of Ribeirão Preto (FFCLRP), University of São Paulo (USP), Ribeirão Preto, SP 14040-901, Brazil; ‡ Department of Biology, Faculty of Philosophy, Sciences and Letters of Ribeirão Preto (FFCLRP), University of São Paulo (USP), Ribeirão Preto, SP 14040-901, Brazil

## Abstract

This study compares 1,3-propanediol (1,3-PDO) production from glycerol, a byproduct of the biodiesel industry, by traditional and electro-driven fermentation. 1,3-PDO is a building block for many polymers and a valuable chemical for various industrial applications. This is the first report about the use of *Pseudomonas aeruginosa* for 1,3-PDO production by electro-fermentation. Electro-fermentation assays were conducted in a single chamber equipped with carbon cloth and platinum electrodes, operated either with a power supply (Δ*V*: 0.01, 0.05, 0.3, or 0.4 V) or under potentiostatic control of 0.4 V vs Ag/AgCl and Cl^–^ (3 mol L^–1^). Compared to traditional fermentation (control), applying different voltages boosted 1,3-PDO production. The highest 1,3-PDO concentration was achieved at 0.05 V: 99.49 ± 0.57 mmol L^–1^, corresponding to a yield of 0.78 ± 0.01 mol of 1,3-PDO mol of glycerol^–1^, a productivity of 15.70 ± 0.25 mmol L^–1^ h^–1^, and a ca. 100% increase compared to the control. Acetic and butyric acid concentrations also increased under electro-fermentation conditions. Electron balance showed that the electro-fermentation favored the reductive pathway for 1,3-PDO formation and that the electron recovery was 89.65 ± 0.37% compared to 45.14 ± 0.89% achieved with traditional fermentation. These findings demonstrate that electro-fermentation is an effective approach to modulate the metabolism of *P. aeruginosa*, enhancing the production of 1,3-PDO. Overall, this work expands the current understanding of the biotechnological potential of *P. aeruginosa* in bioelectrochemical systems and supports the integration of such processes into sustainable biorefineries.

## Graphical abstract

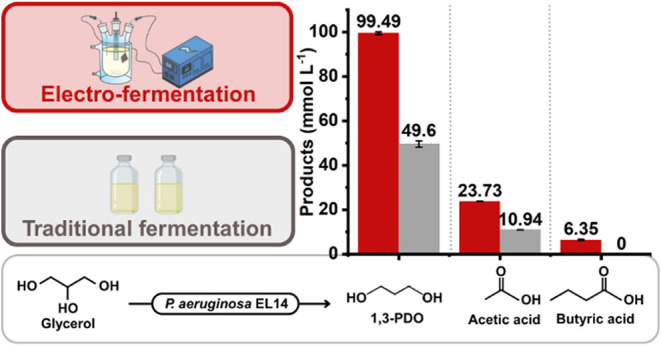

## Introduction

Glycerol bioconversion into 1,3-propanediol (1,3-PDO) represents a strategic approach that uses an industrial byproduct from biorefineries linked to the biodiesel production chain. Indeed, the production of biodiesel, which is widely incorporated into the global energy matrix, generates approximately 10% (w/w) of crude glycerol as a byproduct,^1^ leading to glycerol surplus in the global market.^2^ In this scenario, converting glycerol into higher-value products, such as 1,3-PDO, may be a sustainable solution aligned with the principles of circular bioeconomy. 1,3-PDO is applied in the cosmetic and chemical industries, mainly the polymer industry, and according to Verified Market Reports,^3^ its global market was valued at US$ 594.47 million in 2024 and was projected to reach US$ 1,314.21 million by 2031, with a compound annual growth rate (CAGR) of 11.50%.

Currently, 1,3-PDO is industrially produced via petrochemical routes, by using acrolein or ethylene oxide or through the biotechnological process developed by DuPont, which employs genetically modified *Escherichia coli* strains to convert glucose corn syrup into 1,3-PDO.^4^ However, these routes face sustainability challenges: the former uses petroleum derivatives, while the latter fuels the “food vs fuel” debate.^5^ In this context, glycerol emerges as a sustainable and economically attractive feedstock.

Among the microorganisms that naturally produce 1,3-PDO from glycerol fermentation, notable examples include *Citrobacter freundii*,^6^ *Klebsiella pneumoniae*,^7^ *Clostridium pasteurianum*,^8^ and *Clostridium beijerinckii* ^9^ stand out. In these microbial strains, two metabolic pathways work synergistically to form 1,3-PDO. In the reductive pathway, glycerol dehydratase (GDHt) dehydrates glycerol to 3-hydroxypropionaldehyde (3-HPA), which is followed by NADH+H^+^-dependent 3-HPA reduction to 1,3-PDO mediated by 1,3-propanediol dehydrogenase (1,3-PDODH) to regenerate NAD^+^. Simultaneously, the oxidative pathway generates intermediate compounds, such as dihydroxyacetone (DHA) and pyruvate, regenerating NADH+H^+^. Depending on the microorganism, pyruvate is converted into various organic compounds.^10,11^

Despite the advances achieved in 1,3-PDO production through conventional fermentation, its large-scale application still faces challenges related to low yields and efficiencies, mainly arising from carbon catabolite repression, substrate inhibition, and insufficient NADH+H^+^ regeneration.^12^ In this context, electro-driven fermentation emerges as an innovative strategy to overcome redox balance limitations, enabling the regulation of the NADH+H^+^/NAD^+^ ratio, thereby promoting higher carbon conversion efficiency and reducing the formation of undesired byproducts.^13^

Electro-driven fermentation integrates electrical *stimuli* with traditional fermentation, thereby influencing metabolic pathways and favoring a certain product.^14,15^ This approach is advantageous because it enhances selective bioproduction, uses carbon more efficiently, limits the use of additives for redox balance or pH control, boosts microbial growth, and improves product recovery.^16^ Many electrochemical methods have been employed, including electro-fermentation at constant potential^17,18^ and methods where the electric current is kept constant.^19,20^

Electro-fermentation can alter the oxidation–reduction potential (ORP) of the medium, changing the intracellular ORP and hence the microbial metabolism. Additionally, the electrodes serve as sources or sinks for electrons through extracellular electron transfer (EET) mediated by electroactive bacteria. In both cases, the balance between the electron carriers NADH+H^+^ and NAD^+^ is disturbed, which considerably impacts the direction of the microbial metabolic pathways.^14,21^

In the context of electrodes functioning as electron donors and acceptors, when the final product is more oxidized than the substrate, the working electrode serves as an anode and is used to dissipate excess electrons during anodic electro-fermentation. On the other hand, for a reduced final product, the working electrode functions as a cathode, providing electrons during cathodic electro-fermentation.^22^

During 1,3-PDO production by electro-fermentation, electron supply from a cathode or altered extracellular ORP can increase the reducing power of bacterial metabolism, favoring the reductive pathway. Stimulating the reductive pathway helps to dissipate excess NADH+H^+^ and restore redox homeostasis.

Electro-fermentation systems were initially applied to the production of 1,3-PDO from glycerol fermentation using mixed cultures under the application of an electric current. In these studies, 1,3-PDO production showed a positive correlation with current intensity; however, during long-term operations, the 1,3-PDO-producing microorganisms within the mixed culture were competitively excluded from the biofilm, resulting in a reduced abundance of 1,3-PDO producers and lower final yields. The limitation of microbial succession in mixed cultures can be overcome by using pure 1,3-PDO–producing strains.^4^ Nevertheless, the addition of artificial mediators proved necessary to enhance the interaction between electrodes and microorganisms, with the use of neutral red favoring 1,3-PDO production by *K. pneumoniae*, while Brilliant Blue proved more effective for *C. pasteurianum* DSM.^23,24^

In this context, the use of *P. aeruginosa* in electro-fermentation systems represents a promising alternative due to its electroactive properties, robust biofilm formation, and secretion of natural redox mediators, such as pyoverdine.^25^ In addition, *P. aeruginosa* strains have rarely been described as 1,3-PDO producers and never employed in electro-fermentation for this end. The biocatalyst selected for this study, *P. aeruginosa* strain EL14, isolated in our laboratory, has the ability to produce 1,3-PDO and is electroactive, indicating a promising biocatalyst in electro-fermentation systems for 1,3-PDO production.^26^

The growing 1,3-PDO market demands more competitive biological production methods, positioning glycerol electro-fermentation with *P. aeruginosa* EL14 as a sustainable solution for valorizing biodiesel waste. Unlike conventional systems limited to *Clostridium* and *Klebsiella* cultures, this approach harnesses the organism’s native advantages, including endogenous mediator secretion and biofilm formation, to bypass the need for artificial redox agents.

Therefore, here, we have assessed how electrostimulation affects 1,3-PDO production from glycerol in the presence of *P. aeruginosa* EL14 as a biocatalyst. We have also investigated how the external voltage impacts carbon fate and electron balances during 1,3-PDO production from glycerol. This study contributes to introducing a new promising biocatalyst for 1,3-PDO production and supports the development of sustainable bioprocesses based on bioelectrochemical systems as alternatives to traditional petrochemical routes.

## Experimental Section

### Inoculum

*P. aeruginosa* EL14, isolated by our research group,^26^ was employed as a biocatalyst. This strain was obtained from an exoelectrogenic biofilm formed in a microbial fuel cell (MFC) fed with glycerol.^27^ Its genome is available in GenBank as entry [JASMRB000000000], deposited in the NCBI repository.

*P. aeruginosa* EL14 was preserved in LB medium composed of 10 g L^–1^ tryptone, 5 g L^–1^ yeast extract, and 5 g L^–1^ NaCl, supplemented with 30% glycerol, and stored at −80 °C. To activate *P. aeruginosa* EL14, the stock culture was incubated in 50 mL flasks containing 10 mL of LB medium at 37 °C for 14 h. Then, the preinoculum culture was prepared in 25 mL of the same medium used for fermentation and electro-fermentation and maintained in 50 mL flasks under anaerobic conditions at 37 °C and 150 rpm for 14 h. The inoculum was standardized to an initial optical density at 600 nm (O.D._600_) of 0.2.

### Culture Medium

Fermentation and electro-fermentation were conducted by using M9 minimal medium consisting of 0.25 g L^–1^ MgSO_4_, 1 g L^–1^ NaCl, 1 g L^–1^ NH_4_Cl, 1 g L^–1^ yeast extract, and 100 mmol L^–1^ phosphate buffer. The carbon source was commercial glycerol (xodo Cientfica, 99.5%) at 10 g L^–1^. To remove dissolved oxygen from the medium and headspace, nitrogen gas (grade 5.0) was bubbled for 10 min before the medium was sterilized.

### Fermentation

Traditional batch fermentation assays were performed, as a control and in triplicate, using 100 mL flasks containing 50 mL of M9 minimal medium supplemented with 10 g L^–1^ glycerol. Cultures were incubated at 37 °C under continuous agitation at 150 rpm for 10 h. No electrodes or external voltages were applied in these control assays. Aliquots of 1 mL were collected every 2 h to measure optical density at 600 nm (O.D._600_, BEL Engineering UV-M51 spectrophotometer) and concentrations of glycerol, 1,3-PDO, acetic acid, and butyric acid by high-performance liquid chromatography (HPLC).

### Electro-Fermentation

Electro-bioreactors, custom-made from glass, were used in a single-chamber configuration (125 mL). The electro-fermentation assays were connected to adjustable power supplies (Hikari, HF-3205S) in a two-electrode configuration, separated by a distance of 3 cm. One electrode consisted of a carbon cloth with an area of 9 cm^2^, suspended by a Ni-Cr wire that ensured the external electrical connection. The other electrode was a 9.5 cm long platinum wire with a diameter of 1 mm. The carbon electrode was selected as the working electrode due to its biocompatibility, good electrical conductivity, suitable surface area for biofilm formation, and electrochemical stability. Platinum was used as the counter electrode owing to its high conductivity and chemical inertness, ensuring minimal interference in the system’s redox reactions. A schematic diagram of the setup is provided in the Supporting Information (Figure S1).

The applied voltages (Δ*V*) were 0.01, 0.05, 0.3, and 0.4 V. For this, the negative and positive terminals of the power supply were connected to the platinum and carbon electrodes, respectively. At 0.4 V, electro-fermentation was also conducted by switching the negative terminal to the carbon cloth electrode; this configuration is denoted 0.4 V*. The Δ*V* values were selected to evaluate the system’s response to incremental electrochemical driving forces, ranging from conditions close to equilibrium to higher potentials capable of promoting bioelectrochemical reactions that would not typically occur at small Δ*V*, while avoiding voltages that could trigger undesired side reactions or impair cell viability.

The electro-fermentation assays were carried out at 37 °C and 150 rpm for 10 h in triplicate. Aliquots of 1 mL were collected every 2 h to determine the pH, the O.D._600_ (BEL Engineering spectrophotometer UV-M51), glycerol, 1,3-PDO, and acetic and butyric acid concentrations.

Electro-fermentation experiments were also conducted under controlled potential (potentiostatic electro-fermentation) by using a potentiostat/galvanostat (GAMRY INTERFACE 1010E potentiostat/galvanostat Gamry Instruments). During electro-fermentation with the power supply, an Ag/AgCl, Cl^–^ (3 mol L^–1^ KCl) reference electrode was also integrated into the system to measure the potential of the carbon electrode with a digital multimeter (Digital Multimeter, Hy-5929).

On the basis of the measured potential, new electro-fermentation experiments were conducted under controlled potential. Potentiostatic electro-fermentation was carried out at 0.4 V vs Ag/AgCl, Cl^–^ (3 mol L^–1^ KCl) at 37 °C and 150 rpm for 10 h. Aliquots of 1 mL were collected at the initial time (*t* = 0) and at the end (*t* = 10 h) of electro-fermentation to quantify glycerol, 1,3-PDO, acetic acid, and butyric acid.

### Electrochemical Characterization of Potentiostatic Electro-Fermentation Systems

Electrochemical characterization of the potentiostatic electro-fermentation systems was carried out by using cyclic voltammetry and electrochemical impedance spectroscopy (EIS) with the aid of a GAMRY INTERFACE 1010E potentiostat/galvanostat (Gamry Instruments). All of the analyses were performed at room temperature. M9 medium was employed as a supporting electrolyte, and glycerol (10 g L^–^1) was used as a carbon source.

Cyclic voltammetry analyses were conducted from −0.6 to +0.6 V vs Ag/AgCl and Cl^–^ (3 mol L^–1^ KCl) at a scan rate of 1 mV s^–1^. Voltammetric data were obtained under two experimental conditions: (i) the electro-fermentation bioanode was transferred to a voltammetric cell filled with a fresh and sterile medium, and (ii) a fresh abiotic anode was inserted into the electro-fermentation compartment containing the *P. aeruginosa* E14 planktonic cells. Both measurements were taken at the initial time (*t* = 0) and after electro-fermentation for 6 h, when 1,3-PDO production peaked. This allowed for the mechanisms involved in extracellular electron transfer to be compared.

EIS was carried out at 0.4 V vs Ag/AgCl, Cl^–^ (3 mol L^–1^ KCl) after electro-fermentation for 6 h. The frequency range was 10^–3^ to 10^5^ Hz, and the signal amplitude was 10 mV.

### Kinetic Parameters

The yield of 1,3-PDO produced from glycerol was evaluated on the basis of the conversion factor of the substrate into the product (*Y*_P/S_). *Y*_P/S_ was calculated by using eq 1, where p corresponds to 1,3-PDO (mmol L^–1^), and *s* refers to glycerol (mmol L^–1^).^23^

1 $$Y_{P/S}=\frac{p_{\mathrm{final}}-p_{\mathrm{initial}}}{s_{\mathrm{initial}}-s_{\mathrm{final}}}$$

Productivity (*P*) was calculated by considering the variation in 1,3-PDO concentration (mmol L^–1^, Δ*P*) and bioprocess duration (h, Δ*t*) (eq 2).^28^

2 $$P=\frac{\Delta P}{\Delta t}$$

### Carbon and Electron Balances

The carbon and electron balances (C% and E%, respectively) were estimated in equivalent units by using Cmol for the carbon balance and Emol for the electron balance. For each compound, Cmol and Emol were obtained by multiplying the molar concentration of the compound by its respective carbon (Cequiv) and electron (Eeq) equivalents. The number of Ceq of a compound corresponds to the number of carbon atoms present in its molecule, while the number of Eeq was calculated on the basis of the molecular formula of the compound, as shown in eq 3.^29^

3 $$E_{\mathrm{eq}}\left(C_{w}N_{x}O_{y}H_{z}^{n-}\right)=4w-3x-2y+z+n$$

Table S1 (Supportin-g Information) provides the molecular weights, Ceq, and Eeq for glycerol, 1,3-PDO, acetate, butyrate, and biomass. The *P. aeruginosa* E14 biomass elemental composition was assumed to be CH_1.747_N_0.21_O_0.55_.^30^ The O.D._600_ units were converted into *P. aeruginosa* E14 cell dry weight by using the correlation 1 O.D._600_ = 0.51 g L^–1^.^31^

Finally, C% and E% were calculated by dividing the final Cmol and Emol of each compound by the initial Cmol and Emol, respectively, and multiplying the result by 100 to express the values as percentages (eq 4).^29^

4 $$C\%=\frac{C_{\mathrm{mol}1,3-\mathrm{PDO}}}{C_{\mathrm{mol glycerol}}}\times 100$$

Examples of calculations to estimate carbon and electron recoveries are available in the Supporting Information.

The balances were normalized on the basis of the initial glycerol content without considering the contribution of yeast extract present in the culture medium. Yeast extract is composed of nucleotides, proteins, amino acids, and sugars,^32^ which provide carbon and electrons to metabolism and influence the formation of biomass and organic acids. Therefore, balances exceeding 100% were expected.

### Determination of the Electrochemically Active Area of the Carbon Cloth Electrode

The electrochemical surface area (ECSA) of the carbon cloth electrode was determined in the absence and presence of biofilm according to Almeida et al.^33^ (eq 5).

5 $$\mathrm{ECSA}=\frac{\mathrm{CPE}}{\mathrm{Cs}}$$

where “CPE” is the double-layer capacitance and “Cs” is the graphite specific capacitance (0.0043 mF cm^–2^).

### Quantification of Substrate and Products

Glycerol (substrate) and 1,3-PDO and acetic butyric acid (product) concentrations were determined by high-performance liquid chromatography (HPLC). Analyses were performed on a Shimadzu system comprising an LC-10AD pump, an SIL-20AC HT autosampler, a CBM-20A controller, and a CTO-20AC column oven. The chromatographic column was Aminex HPX-87H (BioRad), maintained at 60 °C. The mobile phase consisted of 5 mmol L^–1^ H_2_SO_4_ at a flow rate of 0.6 mL min^–1^ and a pressure of 84 kgf cm^–2^. Detection was carried out using a refractive index detector (RID-20A). For each analysis, 170 μL of the sample was injected into the chromatograph.

The samples were centrifuged at 9000 rpm for 2 min and filtered through a 0.22 μm membrane filter (PTFE Hydrophilic 0.22 μm—Analítica). The calibration curve was constructed using the six-point calibration method. The concentrations ranged from 0.01 to 15 g L^–1^, and the solutions were prepared with analytical-grade reagents with 99% purity. The calibration curve demonstrated a coefficient of determination (*R* ^2^) of 0.99.

### Statistical Analyses

An analysis of variance (ANOVA) was performed to compare treatment means, followed by Tukey’s multiple comparison test to identify significant differences among experimental groups. ANOVA results were considered significant when *p* was less than 0.05. Tukey’s multiple comparisons were conducted at a 5% significance level (*p* < 0.05). Statistical analyses were performed by using Statistica software, version 14.0.1.

## Results and Discussion

### 1,3-PDO Production

Electro-fermentation (Δ*V* = 0.01, 0.05, 0.3, 0.4, or 0.4* V) increased the 1,3-PDO concentration, yield, and productivity compared to traditional fermentation (TF) (Figure 1). Indeed, TF provided a 1,3-PDO concentration, yield, and productivity of 49.60 ± 1.40 mmol L^–1^, 0.40 ± 0.04 mol of 1,3-PDO mol^–1^ of glycerol, and 8.05 ± 0.27 mmol L^–1^ h^–1^, respectively, whereas electro-fermentation furnished 1,3-PDO concentrations ranging from 86.16 ± 1.29 to 99.49 ± 0.57 mmol L^–1^, yields ranging from 0.65 ± 0.01 to 0.78 ± 0.01 mol of 1,3-PDO mol^–1^ of glycerol, and productivity ranging from 14.82 ± 1.03 to 15.70 ± 0.25 mmol L^–1^ h^–1^ depending on the applied Δ*V*.

**Figure 1:**
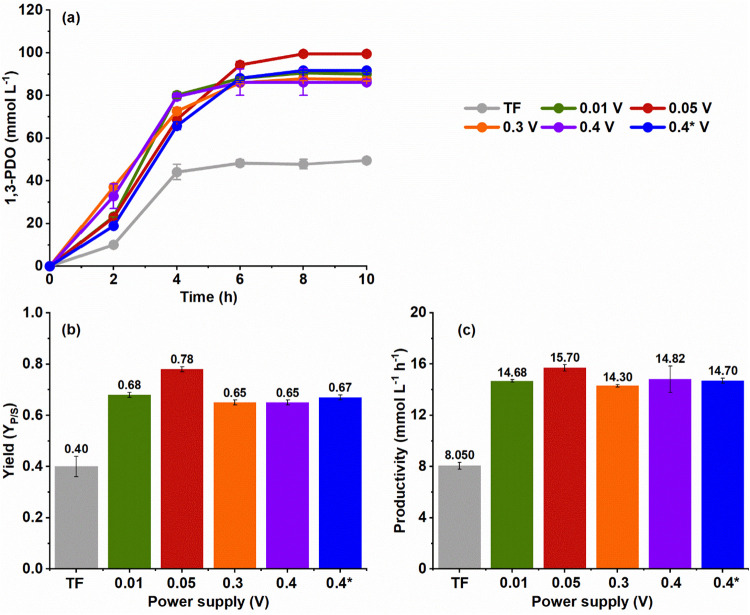
1,3-Propanediol (1,3-PDO) production from glycerol catalyzed by *P. aeruginosa* EL14 during traditional fermentation (TF) or electro-fermentation under different applied Δ*V*. 1,3-PDO (a) concentration, (b) yield (*Y*_P/S_), and (c) productivity. The error bars represent the standard deviation between replicates. Evaluated conditions: TF (gray), 0.01 V (green), 0.05 V (red), 0.3 V (orange), 0.4 V (purple), and 0.4* V (blue).

Among the tested Δ*V* values (0.01–0.4 V), the application of 0.05 V resulted in the highest 1,3-PDO concentration (99.49 ± 0.57 mmol L^–1^), yield (0.78 ± 0.01 mol of 1,3-PDO mol^–1^ of glycerol), and productivity (15.70 ± 0.25 mmol L^–1^ h^–1^), suggesting that this voltage increased the reducing power of the bacterial metabolism, favoring the reductive pathway of glycerol metabolism and consequently enhancing 1,3-PDO biosynthesis by *P. aeruginosa* EL14.

Additionally, there was no significant difference between the electro-fermentation yields at 0.4 V and 0.4* V. Considering that these experiments occurred in a single-chamber cell, the electrode material (carbon or platinum) did not determine polarization and gave similar electron flow. Thus, the electrode material did not influence control of the applied Δ*V*. The choice of carbon cloth as the primary electrode in this study is nevertheless justified by its advantages, including high biocompatibility, which supports the formation of electroactive biofilms, lower cost compared to platinum, and high chemical stability in biological systems.

Table 1 depicts 1,3-PDO production from glycerol electro-fermentation under different electro-fermentation conditions, particularly in the presence of *C. pasteurianum* strains and mixed cultures. Analysis of this table indicates that *P. aeruginosa* EL14 is promising compared with pure bacterial cultures and is competitive compared with mixed cultures. For instance, electro-fermentation in the presence of *K. pneumoniae* L17 at −0.7 V vs Ag/AgCl provided 32.6 mmol L^–1^ 1,3-PDO and a yield of 0.31 mol of 1,3-PDO mol^–1^ of glycerol.^34^ On the other hand, when Zhang et al.^19^ used *C. pasteurianum* G8 as a biocatalyst under −400 mA, they obtained 1585 mmol L^–1^ 1,3-PDO. Although the 1,3-PDO concentration was high in the latter case, the yield was 0.59 mol of 1,3-PDO mol^–1^ of glycerol, within the range of the values presented in Table 1.

**Table 1:** 1,3-Propanediol (1,3-PDO) Production from Glycerol Electro-Fermentation in the Presence of Different Biocatalysts and Conditions^a^

biocatalyst	applied parameter	concentration (**mmol L^–1^)	*Y*_P/S_ (mol mol^–1^)	productivity**** (mmol L^–1^ h^–1^)	references
*Pseudomonas aeruginosa* EL14	0.05 V*	99.5	0.78	15.70	this work
*Clostridium pasteurianum* *G8*	–400 mA	1585	0.59***	63.40	^19^
*Clostridium pasteurianum* *R525*	–0.4 A	78.9	0.11***	2.47	^35^
*Klebsiella pneumoniae* L17	–0.7 V vs Ag/AgCl	32.6	0.31	1.36	^34^
*Escherichia coli* BL21	+0.7 V vs Ag/AgCl	10.4	---	0.32	^18^
*Escherichia coli* BL21	–0.7 V vs Ag/AgCl	16.0	---	0.22	^18^
mixed culture	–0.8 V vs Ag/AgCl	106.0	0.56***	10.60	^36^
mixed culture	–0.9 V vs SHE	4.5	0.50	1.50	^37^
mixed culture	10 A m^–2^	650	0.72	6.10	^20^

a --- Not reported. I: Applied current, V*: Applied voltage, and E: Applied potential. ** The concentrations provided in g L^–1^ were converted to mmol L^–1^. ***Yields of 1,3-PDO originally expressed in g of 1,3-PDO per g of glycerol (g g^–1^) were converted to mol of 1,3-PDO per mol of glycerol (mol mol^–1^). **** The productivity values presented were calculated from the data reported in the respective articles.

Regarding the use of mixed cultures, Zhou et al.^37^ reported a yield of 0.50 mol of 1,3-PDO mol^–1^ of glycerol during electro-fermentation at −0.9 V vs SHE, while Roume et al.^20^ achieved 0.72 mol of 1,3-PDO mol^–1^ of glycerol under 10 A m^–2^.

Productivity of 1,3-PDO reported in the literature varies significantly depending on the microorganism used, electrochemical conditions, and operational mode employed (Table 1). In the present study, *P. aeruginosa* EL14 had a productivity of 15.70 mmol L^–1^ h^–1^, surpassing values obtained with pure cultures of *K. pneumoniae* (1.36 mmol L^–1^ h^–1^),^34^ *C. pasteurianum* (2.47 mmol L^–1^ h^–1^),^35^ and *E. coli* (0.22–0.32 mmol L^–1^ h^–1^),^18^ as well as previously described mixed cultures (6.0–10.0 mmol L^–1^ h^–1^).^20,36,37^ The highest reported productivity of 63.4 mmol L^–1^ h^–1^ was achieved by *C. pasteurianum* G8 in fed-batch mode.^19^ This operational strategy is particularly advantageous as it allows controlled glycerol replenishment, preventing both substrate inhibition (due to excess) and limitation (due to depletion). Consequently, the production phase is prolonged, optimizing the microbial performance. These conditions explain the exceptional productivity observed in this case.

These results not only highlight that *P. aeruginosa* EL14 is an effective, unusual biocatalyst for glycerol electro-fermentation to 1,3-PDO but also underscore the potential use of pure cultures in bioelectrochemical systems. While mixed cultures often offer metabolic flexibility, pure cultures provide greater control and reproducibility, which are essential for obtaining reproducible results in bioelectrochemical processes.

### Organic Acid Production

Besides 1,3-PDO, acetic and butyric acids emerged during glycerol fermentation catalyzed by *P. aeruginosa* EL14.^26^ Electro-fermentation resulted in an increase in the acetic acid concentration compared to TF (10.94 ± 0.03 mmol L^–1^). The concentrations varied according to the applied Δ*V*, with a significant difference between the conditions of 0.3 V and 0.4* V, which resulted in 19.97 ± 0.15 and 26.46 ± 0.17 mmol L^–1^, respectively (Figure 2a).

**Figure 2:**
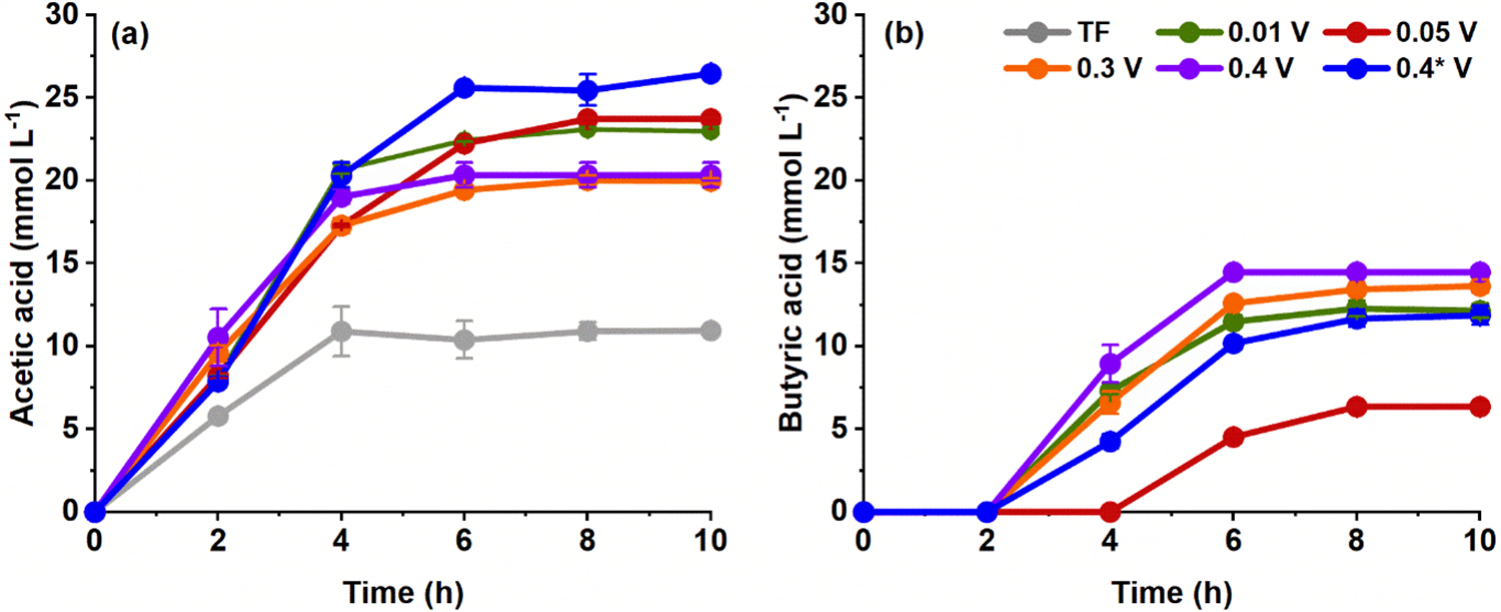
(a) Acetic and (b) butyric acid concentrations over time during 1,3-propanediol production from glycerol catalyzed by *P. aeruginosa* EL14 by traditional fermentation (TF) or electro-fermentation under different applied Δ*V*. Error bars represent the standard deviation between replicates. Evaluated conditions: TF (gray), 0.01 V (green), 0.05 V (red), 0.3 V (orange), 0.4 V (purple), and 0.4* V (blue).

Butyric acid was detected only under electro-fermentation conditions, with concentrations ranging from 6.35 ± 0.30 to 14.47 ± 0.12 mmol L^–1^, depending on the applied Δ*V* (Figure 2b). Cardeña et al.^14^ reported similar results. They found that, compared to TF, the metabolic profile changed during electro-fermentation. Indeed, the authors detected ethanol production in the presence of mixed cultures under electrochemical conditions, but not during TF. These findings suggest that polarized electrodes influence the ORP of the fermentation medium and microbial metabolism to give products not typically detected under TF conditions.^38^

The fact that electro-fermentation furnished higher organic acid concentrations than TF may be associated with the need to maintain the redox balance as the 1,3-PDO production increases. The metabolic pathway giving 1,3-PDO is reductive and consumes NADH+H^+^ while forming NAD^+^, whereas the oxidative pathway leading to organic acid formation consumes NAD^+^ and regenerates NADH+H^+^. When electrodes alter the extracellular ORP, the intracellular ORP is directly affected. Given that controlling redox homeostasis is essential for metabolic function, changes in extracellular ORP trigger adjustments in intracellular electron flows, modifying the ratio between NADH+H+ and NAD+. This allows metabolic pathways to be controlled by directing them toward specific products.^21^ In other words, the reductive branch stimulates the oxidative branch of metabolism.

Therefore, increased 1,3-PDO production during electro-fermentation suggests enhanced activity of the oxidative pathway, with bacterial growth and organic acid formation. This balances the consumption and regeneration of reducing equivalents, thereby maintaining redox homeostasis.

It is important to note that changes in the polarization of the electrodes (0.4 and 0.4 V*) in the single-chamber system configurations did not affect the 1,3-PDO production; however, differences in the distribution of acetic and butyric acids were detected. These may result from metabolic adjustments in response to changes in electrode polarity (i.e., which electrode is positive or negative), which modulate the extracellular redox environment and thus intracellular redox homeostasis. Both configurations yielded similar electrochemical conditions.

### *P. aeruginosa* E14 Cell Growth and Glycerol Consumption

Another aspect to consider is the *P. aeruginosa* E14 population growth (Figure 3a). The maximum O.D._600_ during TF was 2.14 ± 0.04. In contrast, during the electro-fermentation assays, *P. aeruginosa* E14 growth was higher and varied with the applied Δ*V*. The highest O.D._600_ was achieved at 0.4 and 0.4* V (2.58 ± 0.02 and 2.63 ± 0.03, respectively). These data suggest that electro-fermentation favored *P. aeruginosa* E14 population growth, probably because glycerol was more efficiently used during metabolism.

**Figure 3:**
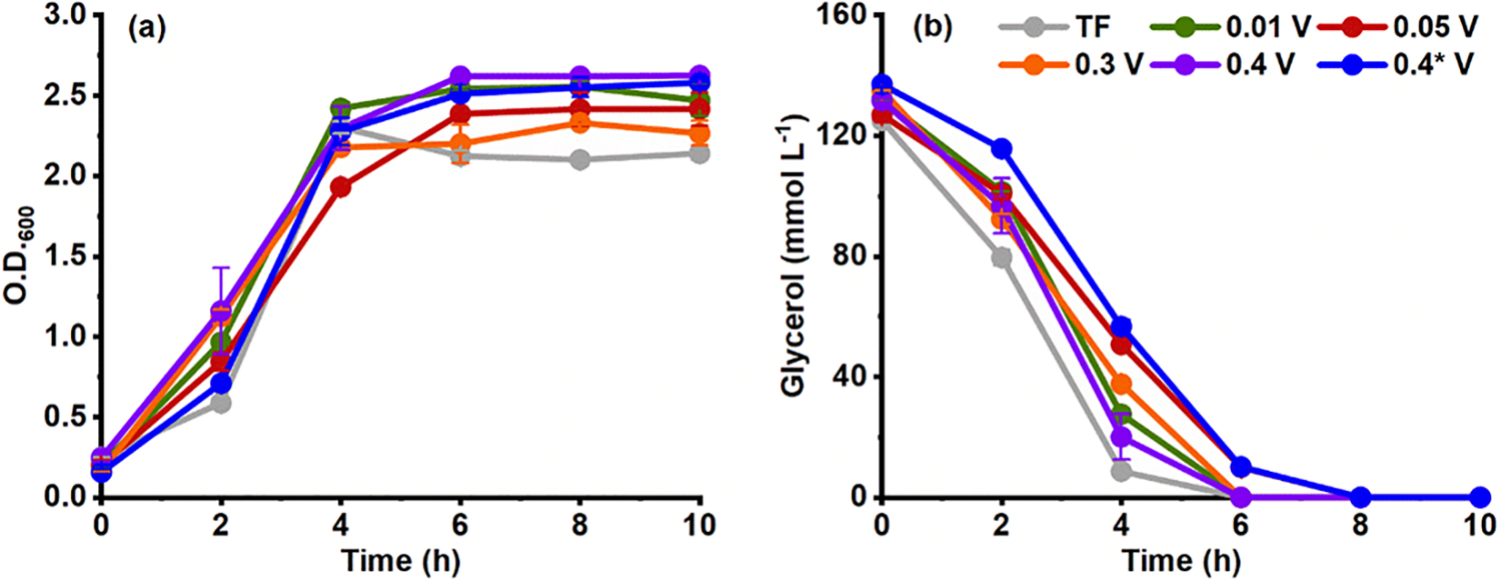
(a) *P. aeruginosa* EL14 population growth and (b) glycerol consumption during 1,3-propanediol production from glycerol catalyzed by *P. aeruginosa* EL14 by traditional fermentation (TF) or electro-fermentation under different applied Δ*V*. The error bars represent the standard deviation between replicates. Evaluated conditions: TF (gray), 0.01 V (green), 0.05 V (red), 0.3 V (orange), 0.4 V (purple), and 0.4* V (blue).

In all of the assays, *P. aeruginosa* EL14 completely consumed the available glycerol within 6 to 8 h (Figure 3b).

In electro-fermentation, the application of a Δ*V* of 0.05 V resulted in the best conditions for 1,3-PDO production in the presence of *P. aeruginosa* EL14 in terms of 1,3-PDO concentration, yield, and productivity. However, this condition did not provide the highest *P. aeruginosa* EL14 population growth. This suggests that increased 1,3-PDO production is more related to the electrostimulation of fermentative pathways rather than the number of *P. aeruginosa* E14 cells. Corroborating this hypothesis, no significant differences in O.D.600 were observed between the different Δ*V*. Therefore, an applied Δ*V* of 0.05 V redirected the metabolism toward efficient glycerol use for 1,3-PDO production instead of biomass formation.

### Electron and Carbon Balances

During TF, 45.14 ± 0.89% and 39.65 ± 0.89% of electrons and carbon were recovered during 1,3-PDO production, respectively. During electro-fermentations, recoveries increased: electron recovery ranged from 74.08 ± 0.42% to 89.65 ± 0.37%, while carbon recovery varied between 64.82 ± 0.42% and 78.44 ± 0.37% for applied Δ*V* of 0.3 and 0.05 V, respectively (Figure 4). This reflects that the reducing power and carbon flow were redirected toward 1,3-PDO production, highlighting that electro-fermentation promoted metabolism toward this product.

**Figure 4:**
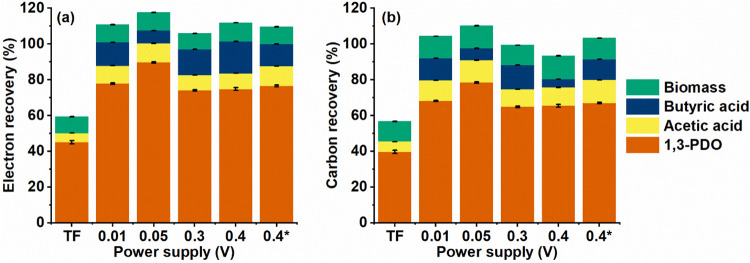
(a) Electron and (b) carbon balances for 1,3-propanediol (1,3-PDO) production from glycerol catalyzed by *P. aeruginosa* EL14 by traditional fermentation (TF) or electro-fermentation under different applied Δ*V*. Error bars represent the standard deviation among replicates. Results are normalized on the basis of the electron content of the initial glycerol mass. Electron and carbon recoveries for cell biomass are colored green, butyric acid blue, acetic acid yellow, and 1,3-PDO is represented in orange.

Electro-fermentation also redistributed the electron and carbon flows toward the oxidative pathways. During TF, only 5.00 ± 0.05% of electrons and 5.83 ± 0.05% of carbon were allocated to organic acid formation. In turn, during electro-fermentation, these values increased and reached 26.52 ± 0.07% of electrons and 24.45 ± 0.06% of carbon at 0.4 and 0.4* V, respectively. These results are associated with higher acetic and butyric acid production, demonstrating that electrode polarization favored both the reductive and oxidative pathways. Metabolic adaptation is essential for maintaining cellular redox balance.

Among the tested conditions, electro-fermentation at 0.05 V resulted in the highest carbon (78.44 ± 0.37%) and electron (89.65 ± 0.37%) recoveries for 1,3-PDO production. This performance was accompanied by increased flows toward the oxidative pathway, with 19.15 ± 0.025% of carbon and 17.84 ± 0.025% of electrons allocated to organic acid synthesis. These results indicate that an applied Δ*V* of 0.05 V improved the metabolic balance between the reductive and oxidative pathways, maximizing glycerol conversion into 1,3-PDO and thus representing the most favorable condition for 1,3-PDO production by *P. aeruginosa* EL14.

### Electro-Fermentation under Potentiostatic Control

Different modes exist to control electro-fermentation systems.^38^ Traditionally, a potential or current controlled by a potentiostat is applied.^18,19^ Here, we also implemented electro-fermentation under potentiostatic control. For this, we first measured the potential of the carbon cloth electrode against a reference electrode during voltage control assays. The best electro-fermentation condition was applied Δ*V* of 0.05 V, which corresponds to the carbon cloth working electrode of 0.4 V vs Ag/AgCl, Cl^–^ (3 mol L^–1^). At this potential, the 1,3-PDO concentration was 96.72 ± 0.61 mmol L^–1^, and the yield was 0.74 ± 0.05 mol of 1,3-PDO mol^–1^ of glycerol (Figure 5). Figure 6 also confirms that these values are similar compared to the results obtained under a polarization of 0.05 V, where the concentration of 1,3-PDO was 99.49 ± 0.57 mmol L^–1^ and the yield was 0.78 ± 0.01 mol of 1,3-PDO mol^–1^ of glycerol. These results confirm the robustness of the experimental method using a power supply, supporting its use as an alternative to the potentiostat in electro-fermentation experiments.

**Figure 5:**
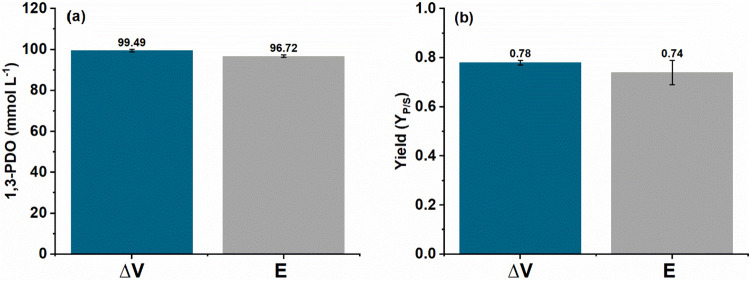
1,3-PDO concentration (a) and yield (b) of 1,3-propanediol (1,3-PDO) during electro-fermentation using the two-electrode configuration (power supply, Δ*V* = 0.05 V/ blue) and under the potentiostatic mode (*E* = 0.4 V vs Ag/AgCl, Cl^–^ (3 mol L^–1^)/ gray).

**Figure 6:**
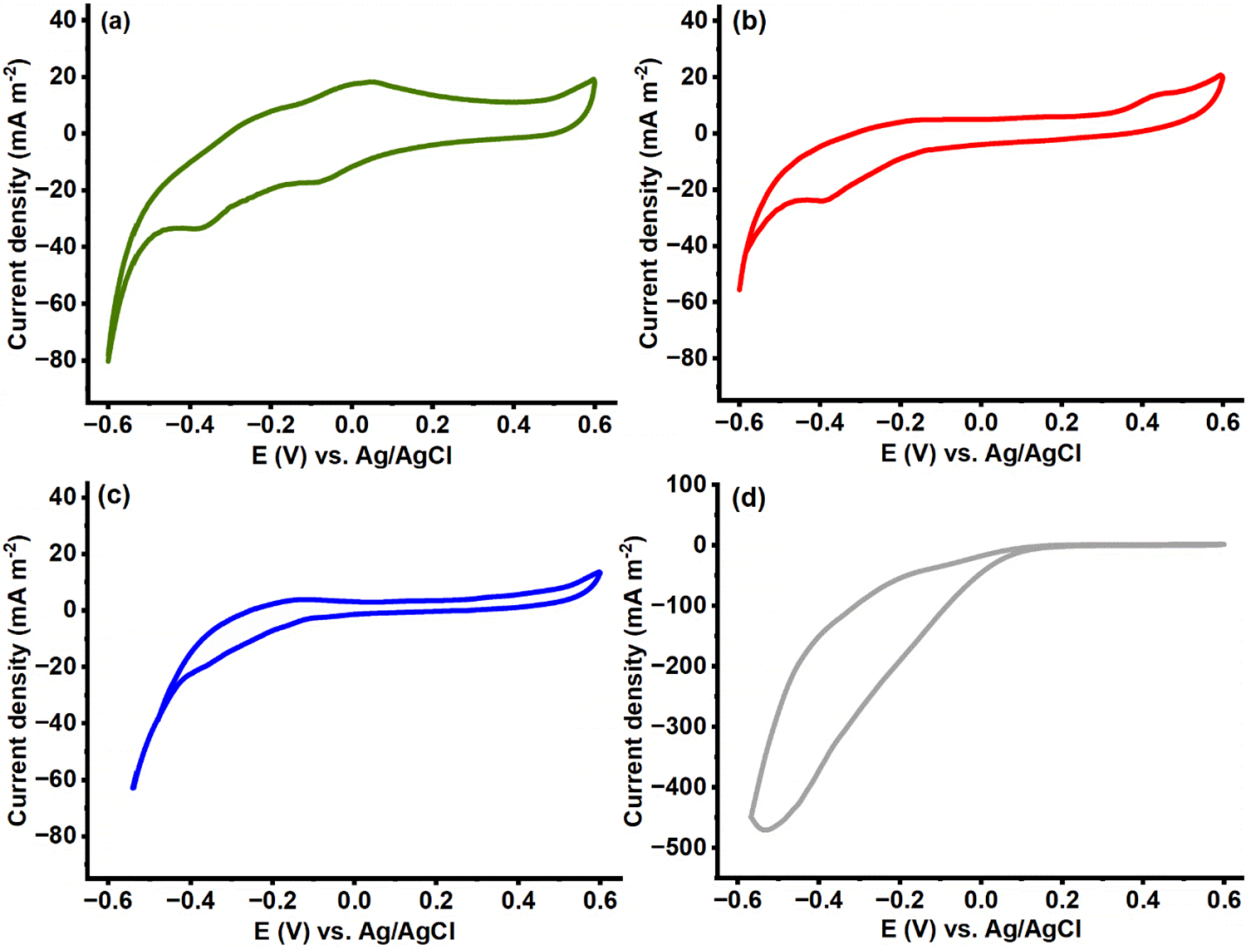
Voltammograms obtained at different stages of glycerol electro-fermentation catalyzed by *P. aeruginosa* EL14 at a scan rate of 1 mV s^–1^. The curves were recorded at (a) *t* = 0, (b) *t* = 6 h, and (c) after the replacement of the working electrode by a sterile one. The blank voltammogram is shown in part (d).

### Electrochemical Characterization

We carried out electrochemical characterization during potentiostatic electro-fermentation at 0.4 V vs Ag/AgCl, Cl^–^ (3 mol L^–1^) and performed cyclic voltammetry under two conditions to investigate the electrochemical activity and possible EET mechanism (Figure 6). Fresh inoculum solution (Figure 6a) displayed one oxidation peak, at 0.032 V (EpA1) vs Ag/AgCl, Cl^–^ (3 mol L^–1^), and two reduction peaks, at −0.091 V (EpC1) and −0.38 V (EpC2) vs Ag/AgCl, Cl^–^ (3 mol L^–1^), which indicate the presence of redox-active compounds associated with the initial electrochemical processes.

After electro-fermentation for 6 h (Figure 6b), the electrochemical profile changed. The oxidation and reduction peaks initially observed at 0.032 V and −0.091 V vs Ag/AgCl, Cl^–^ (3 mol L^–1^) were suppressed, an oxidation peak appeared at 0.44 V vs Ag/AgCl, Cl^–^ (3 mol L^–1^) (EpA2), and the reduction peak at −0.38 V vs Ag/AgCl, Cl^–^ (3 mol L^–1^) (EpC2) remained. These changes suggest a transition in the redox compounds throughout the electro-fermentation process. The absence of the initial peaks indicates that the compounds underlying the reactions were consumed during the early phase of electro-fermentation. The appearance of an oxidation peak at 0.44 V vs Ag/AgCl, Cl^–^ (3 mol L^–1^) suggests that new redox species emerged or that a *P. aeruginosa* EL14 biofilm emerged on the electrode surface. To confirm this hypothesis, we replaced the working electrode with a new sterile electrode (Figure 6c). The absence of redox activity on the new electrode indicates that the electrochemical activity observed after electro-fermentation for 6 h was directly associated with *P. aeruginosa* EL14 biofilm formation on the electrode surface. The oxidation peak observed at 0.44 V in the biofilm-associated voltammetry but absent in the supernatant suggests the involvement of redox-active compounds, which could be retained within the extracellular polymeric substance (EPS) matrix or anchored to the cell surface, rather than soluble metabolites diffused into the medium.

Previous studies have demonstrated that concentration and potential gradients across the EPS matrix exert a significant influence on electron transfer processes. Various EPS constituents, including extracellular DNA, humic acids, and certain proteins, exhibit intrinsic redox activity or conductive/semiconductive properties.^39^ EPS can act as a reservoir for electroactive substances (e.g., flavins and c-type cytochromes and Fe-S proteins), serving as an electron transport medium. This arrangement enables EPS-embedded cells to transfer electrons to external acceptors or receive electrons from donors, with electron hopping being a plausible molecular mechanism for such conduction.^40^

In this context, it is plausible that the electroactive species detected in this study is part of this EPS-associated redox network and acts as a mediator in extracellular electron transfer within our electro-fermentation system.

The blank voltammogram (Figure 6d) did not exhibit any redox peaks, which reinforced that the electrochemical activity observed in Figure 6a–c arose from *P. aeruginosa* EL14 metabolism and biofilm formation. This finding supports the hypothesis that the biofilm enables extracellular electron transfer, which is essential for electrostimulation processes. In such systems, the microbial biofilm acts as an electroactive interface, mediating electron flow between the electrode and cellular metabolism, thereby influencing metabolic pathways involved in glycerol conversion, as observed in the electro-fermentation assays performed in this study.

The oxidation peak at 0.44 V vs Ag/AgCl, Cl^–^ (3 mol L^–1^) (Figure 6b) is notably close to the electrochemical potential applied during potentiostatic electro-fermentation (0.4 V vs Ag/AgCl, Cl^–^ (3 mol L^–1^)). This suggests that EET occurred during electro-fermentation. Additionally, the reduction peak around −0.38 V vs Ag/AgCl, Cl^–^ (3 mol L^–1^) indicates that *P. aeruginosa* EL14 likely supplied electrons from the cathode (counter electrode). These results suggest that, during electro-fermentation at 0.4 V vs Ag/AgCl, Cl^–^ (3 mol L^–1^), *P. aeruginosa* EL14 carried out bidirectional electron transfer, donating electrons to the anode (working electrode, carbon) and accepting electrons from the cathode (counter electrode). These findings indicate that the increased concentration and yield obtained during electro-fermentation at 0.05 V may be related to the EET contribution to enhancing the reducing power of bacterial metabolism. This is particularly relevant considering that 1,3-PDO biosynthesis follows a reductive pathway that depends on reducing equivalents such as NADH+H^+^. Thus, the additional input of electrons from the cathode may have favored the intracellular redox balance, promoting NADH+H^+^ regeneration and consequently driving carbon and electron flux toward 1,3-PDO production.

Figure 7 presents the Nyquist diagrams obtained from EIS measurements performed at 0.4 V vs Ag/AgCl. As expected, there is a huge decrease of the charge transfer resistance inferred from the *Z*-value (semicircle diameter) at lower frequencies in the presence of the *P. aeruginosa* EL14 when compared to the abiotic system. The employed equivalent circuit (EC), composed of two RC loops in series, incorporates a resistor (Rct), a constant phase element (CPE), and a mass-transfer-related element (restricted linear diffusion (M) or anomalous diffusion (Ma)), as shown in Figure 7c. The obtained fitted values are shown in Tables S2 and S3.

**Figure 7:**
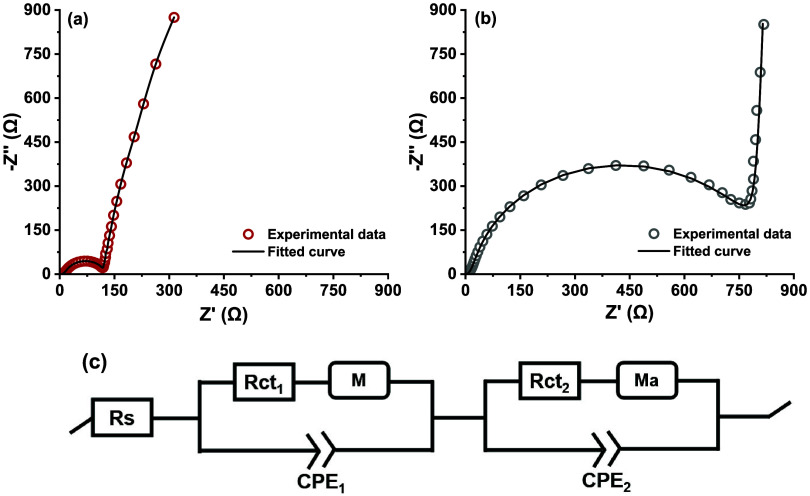
Nyquist diagram of the impedance spectra obtained at *t* = 6 h of glycerol electro-fermentation catalyzed by *P. aeruginosa* EL14 (a) or in the abiotic system (b), measured in a frequency (black square) range of 0.001 to 10^5^ Hz with an amplitude of 10 mV. Impedance measurements were performed at 0.4 V vs Ag/AgCl, Cl^–^ (3 mol L^–1^). (c) Equivalent circuit used to fit the measured impedance spectra.

The two loops of the equivalent circuit reflect distinct electrochemical processes occurring at low and high frequencies, corresponding to the two-phase angle peaks observed in the Bode plot (Figure S2). The presence of multiple peaks in the Bode plot or semicircles in the Nyquist diagram has been reported for bioelectrochemical systems in microbial fuel cells.^41^

Elements Rct_1_, M1, and CPE_1_ predominantly describe the electron transfer mainly at the electrode/electrolyte interface. Elements Rct_2_, Ma, and CPE_2_ can be associated with the porous electrode structure or the biofilm matrix, reflecting diffusional and kinetic limitations. The inclusion of the anomalous diffusion element replacing the classical Warburg element reflects the complexity of the interface. As discussed by Sanchez-Herrera et al.,^42^ anomalous diffusion arises in systems with complex geometries or spatial constraints, such as those imposed by the biofilm matrix. In the abiotic system, anomalous diffusion (characterized by diffusion resistance (Rd) of 6.66 × 10^–3^ Ω and diffusion time (td) of 0.15 × 10^–6^ s) indicates the presence of nonconventional diffusional processes even in the absence of biofilm. This behavior may be related to the intrinsic structure of the carbon cloth, whose porosity creates heterogeneous diffusion pathways. Similar behavior was reported by Cao et al.,^43^ where mass transfer was limited by the porous structure of carbon fiber electrodes, resulting in Nyquist diagrams exhibiting an almost vertical line in the low-frequency region, a characteristic of porous surfaces.

In the presence of the *P. aeruginosa* EL14 biofilm, the anomalous diffusion parameter (Ma) changed to Rd 1.27 × 10^–3^ Ω and td 1.01 s. The increased diffusion time reflects the spatial constraints imposed by the biofilm extracellular matrix, whose complex three-dimensional structure, composed of extracellular polymers and microbial cells, creates a heterogeneous transport environment. Despite this greater spatial restriction, the reduction in Rd indicates that the direct electron transfer mechanism (Figure 6b,6c) overcomes the diffusional barrier, establishing preferential pathways for electron flow within the biofilm.^44^

The abiotic system exhibited a high charge transfer resistance (Rct_1_) (783.60 Ω), which has been reduced to 12.88 Ω in the presence of the biofilm, corroborating the role of the biofilm as an electron transfer facilitator. Conversely, Rct_2_ increased from 35.80 Ω in the abiotic system to 103.30 Ω with the biofilm, indicating that although the biofilm functions as an electronic conductor, it also introduces kinetic limitations associated with substrate diffusion or impedance generated by its extracellular matrix.^45^

The total charge transfer resistance (Rct) was determined as the sum of elements Rct_1_ and Rct_2_ from the equivalent circuit, resulting in 819.43 ± 2.96 Ω for the abiotic system and 116.18 ± 0.52 Ω in the presence of the *P. aeruginosa* EL14 biofilm. These values corroborate the observation that the semicircle diameter decreased considerably in the presence of the biofilm, as evidenced by the simplified circle fitting method, which yielded Rct values of 883.37 and 120.92 Ω for the abiotic and biotic systems, respectively.

The electrochemical behavior observed indicates that the *P. aeruginosa* EL14 biofilm predominantly acted as an electron conductor rather than as a capacitor. This interpretation is supported by the significant reduction in charge transfer resistance (Rct), accompanied by modest variations in parameters related to the capacitive response, such as the constant phase element (CPE, obtained from circle fitting) and electrochemically active surface area (ECSA). CPE decreased from 9.98 μF in the abiotic system to 8.63 μF in the presence of the biofilm. The decrease in CPE, which reflects a reduction in the area available for charge accumulation in the double layer, suggests that while biofilm formation partially restricted this area, the predominant effect was the facilitation of electron transfer, contributing to the improvement in the electrochemical conductivity of the system. This behavior was also reported by Kim et al.,^46^ who observed reduced capacitance values after the development of electroactive biofilms. Consistently, the ECSA values ranged from 2.32 cm^2^ (abiotic) to 2.01 cm^2^ (biotic), suggesting that biofilm formation did not substantially alter the electrochemically active surface area of the system. Collectively, these data indicate that biofilm formation facilitated electron transfer, reinforcing its contribution to the improvement of the system’s electrochemical conductivity.

The formation of a highly conductive *P. aeruginosa* EL14 biofilm corroborates the enhanced performance observed under electro-fermentation at 0.05 V using a power supply, a condition equivalent to 0.4 V at the carbon electrode in the potentiostatic system, when compared to the other applied voltages. The combination of ORP modulation and electron uptake by the biofilm likely increased the availability of NADH+H^+^ (i.e., reducing power), thereby favoring 1,3-PDO production. The interplay between ORP control and enhanced electron transfer underscores the importance of both processes in driving 1,3-PDO synthesis, suggesting that these mechanisms act synergistically during *P. aeruginosa* EL14 catalyzed glycerol electro-fermentation.

## Conclusions

This study demonstrates, for the first time, that *P. aeruginosa* EL14 can serve as an effective biocatalyst for 1,3-propanediol (1,3-PDO) production from glycerol in electro-fermentation systems. The application of electrical stimuli significantly enhanced 1,3-PDO yield, particularly at 0.05 V (99.49 ± 0.57 mmol L^–1^ 1,3-PDO and 0.78 ± 0.01 mol 1,3-PDO mol^–1^ glycerol), which is likely associated with improved extracellular electron transfer (EET) dynamics that increase the reducing power of the bacterial metabolism. Additionally, increased concentrations of organic acids were observed during electro-fermentation, suggesting metabolic adjustments to maintain redox homeostasis. These findings highlight the potential of electro-fermentation to enhance 1,3-PDO production catalyzed by *P. aeruginosa* EL14 and underscore the value of this technology for producing high-value compounds from biofuel industry byproducts. This approach supports the integration of bioelectrochemical processes into sustainable biorefineries, offering a promising alternative to conventional petrochemical routes.

## Supplementary Material



## Notes

Schematic diagram of the electro-fermentation experimental setup with adjustable power supply and table of molar mass; carbon equivalents; electron equivalents of glycerol metabolism compounds; bode plots of the impedance spectra measured during the electro-fermentation experiments; along with the equivalent circuit fitting parameters for electrochemical impedance spectroscopy, and including both the abiotic control system and the system with *P. aeruginosa* EL14 biofilm (PDF)

## Abbreviations

ΔV: voltage
TF: traditional fermentation
EET: extracellular electron transfer
