# Supplementary material for: Unlocking 1,3-Propanediol Production by Pseudomonas aeruginosa through Electro-Fermentation
Source: ACS Omega. 2025 Sep 22;10(39):45438–49. doi: 10.1021/acsomega.5c05374 (PMC12509004; doi:10.1021/acsomega.5c05374)
Supplement: Supplementary file 1 [file ao5c05374_si_001.pdf]

Unlocking 1,3-propanediol production by *Pseudomonas aeruginosa* through electro-fermentation

Julia Pereira Narcizo[†], María-Eugenia Guazzaroni[‡], Adalgisa Rodrigues de Andrade[†], Valeria Reginatto[†*]

[†]Department of Chemistry, Faculty of Philosophy, Sciences and Letters of Ribeirão Preto (FFCLRP), University of São Paulo (USP), Ribeirão Preto, SP, 14040-901, Brazil

[‡]Department of Biology, Faculty of Philosophy, Sciences and Letters of Ribeirão Preto (FFCLRP), University of São Paulo (USP), Ribeirão Preto, SP, 14040-901, Brazil

*Corresponding Author:

E-mail: valeriars@ffclrp.usp.br

Phone: +55 (16) 3315-0431

Supplementary Materials

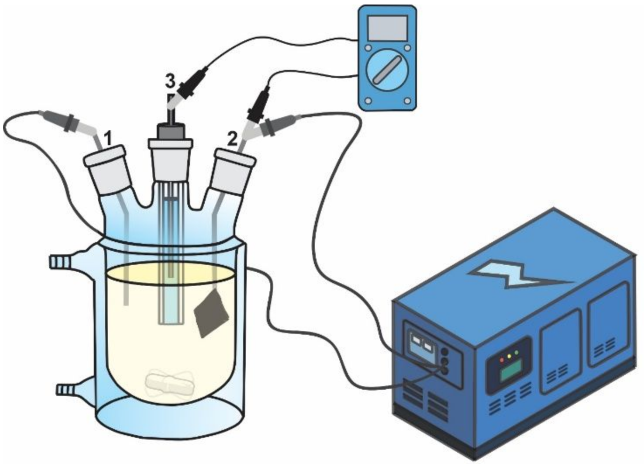

Figure S1. Schematic representation of the electro-fermentation experimental setup conducted by applying and external voltage and using an adjustable power supply. 1 - platinum electrode; 2 - carbon cloth electrode suspended by a Ni-Cr wire; 3 - Ag/AgCl,Cl- (3 mol L-1 KCl) reference electrode. The potential of the carbon electrode was measured with a digital multimeter.

Table S1. Molar mass, carbon equivalents, and electron equivalents of various compounds from glycerol metabolism.

Compound	Molecular formula	Molecular weight (g mol^{-1})	C_{eq}	E_{eq}
Acetate	$C_2H_3O_2^-$	59.04	2	8
Glycerol	$C_3H_8O_3$	92.08	3	14
1,3-PDO	$C_3H_8O_2$	76.10	3	16

Biomass *P. aeruginosa*	$CH_{1,747}N_{0,21}O_{0,55}$	25.49	1	3.74
Butyrate	$C_4H_7O_2^-$	87.10	4	20

As an example of a calculation for estimating carbon recovery, considering an initial glycerol concentration of 5 g/L, the initial carbon content (Cmol initial) would be:

$$\frac{(5\ ^{g}/_{L}) * 3}{92.08\ ^{g}/_{mol}} = 0.16\ \text{Cmol}$$

If at the end of the bioprocess the 1,3-PDO concentration is 0.5 g/L, the corresponding final carbon content (Cmol final) would be:

$$\frac{(0.5\ ^{g}/_{L}) * 3}{76.10\ ^{g}/_{mol}} = 0.020\ \text{Cmol}$$

Therefore, the fraction of the initial carbon recovered as 1,3-PDO would be:

$$\left(\frac{0.020\ Cmol}{0.16\ Cmol}\right) * 100 = 12.50\%$$

A similar calculation could be performed to estimate the percentage of initial electron recovery, resulting in 14.47% as 1,3-PDO.

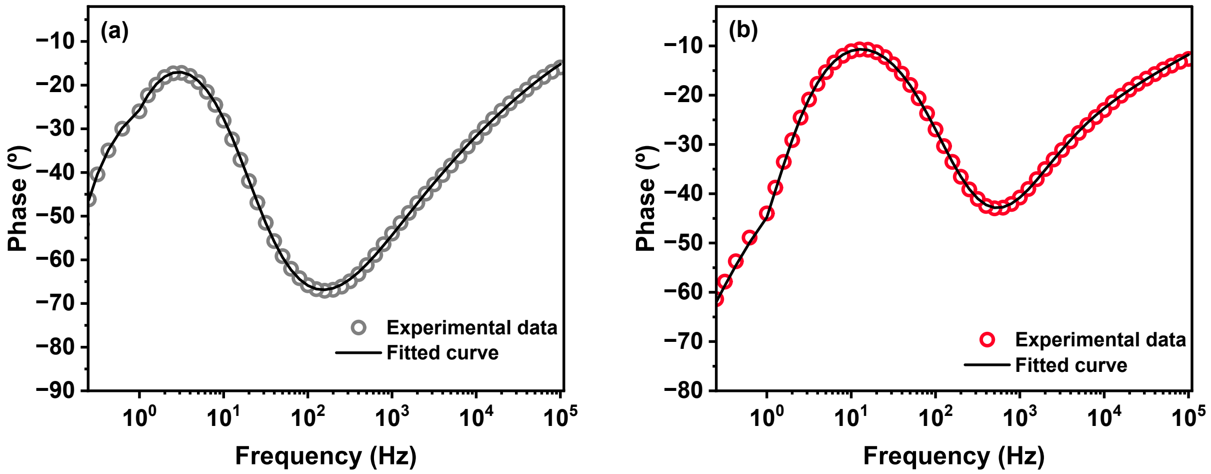

Figure S2. Bode plots of impedance spectra measured at t = 6 h of glycerol electro-fermentation catalyzed by *P. aeruginosa* EL14 (b) or in the abiotic system (a). Measurements were performed in a frequency range of 0.001 to 105 Hz with an amplitude of 10 mV at 0.4 V vs. Ag/AgCl.

Table S2. Equivalent circuit fitting parameters for electrochemical impedance spectroscopy of the abiotic system.

Parameter	Value	Unit	Std. Error
Rs	4.25	Ω	0.032
Rct_1	783.60	Ω	2.71
RdM	0.27	Ω	2.84×10^{-3}
tdM	0.14×10^{-3}	s	1.46×10^{-6}
CPE_1	13.10×10^{-6}	$F.s^{a-1}$	74.12×10^{-9}
a_1	0.94		1.27×10^{-3}
Rct_2	35.83	Ω	1.19

RdMa	6.66 x 10^{-3}	Ω	0.58 x 10^{-3}
tdMa	0.15 x 10^{-6}	s	27.54 x 10^{-9}
aMa	0.62		3.27 x 10^{-3}
CPE_2	0.77 x 10^{-3}		5.68 x 10^{-6}
a_2	0.480	$F.s^{a-1}$	0.590 x 10^{-3}
X^2	3.01 x 10^{-3}		

Table S3. Equivalent circuit fitting parameters for electrochemical impedance spectroscopy of the system with *Pseudomonas aeruginosa* EL14 biofilm.

Parameter	Value	Unit	Std. Error
Rs	6.71	Ω	0.059
Rct_1	12.88	Ω	0.30
RdM	2.19	Ω	0.024
tdM	2.34 x 10^{-3}	s	28.58x10^{-6}
CPE_1	0.41 x 10^{-3}	$F.s^{a-1}$	8.594x10^{-6}
a_1	0.51		1.099x10^{-3}
Rct_2	103.30	Ω	0.43
RdMa	1.27 x 10^{-3}	Ω	2.29x10^{-5}
tdMa	1.011	s	0.65 x 10^{-3}
aMa	0.039		0.0012
CPE_2	25.15 x 10^{-6}		0.30x10^{-6}
a_2	0.873	$F.s^{a-1}$	2.12 x 10^{-3}
X^2	4.45 x 10^{-3}		
